# Modified surgical anchor refixation in older patients with acute proximal hamstring rupture: clinical outcome, patient satisfaction and muscle strength

**DOI:** 10.1007/s00402-022-04752-3

**Published:** 2023-01-09

**Authors:** Tomáš Chocholáč, Linda Bühl, Corina Nüesch, Niklas Bleichner, Annegret Mündermann, Karl Stoffel

**Affiliations:** 1 Department of Orthopaedics and Traumatology, University Hospital Basel, Spitalstrasse 21, 4031 Basel, Switzerland; 2 Department of Biomedical Engineering, University of Basel, Basel, Switzerland; 3 Department of Spine Surgery, University Hospital Basel, Basel, Switzerland; 4 Department of Clinical Research, University of Basel, Basel, Switzerland

**Keywords:** Proximal hamstring rupture, Surgical repair, Modified anchor placement, Patient satisfaction

## Abstract

**Introduction:**

After conventional surgical refixation of the hamstrings after proximal hamstring rupture, patients frequently experience pain while sitting and deficits in hamstring muscle strength of the operated side. To improve these outcomes, we have modified the surgical anchor placement and have carried out a thorough follow-up examination.

**Materials and methods:**

Thirteen older patients (8 female, 5 males) with a median age of 64.2 (range, 52.1–80.4) years were surgically treated for acute proximal hamstring rupture using modified anchor placement and participated in a follow-up assessment at a median of 46.2 (11.2–75.0) months after surgery. Patients completed the Perth Hamstring Assessment Tool (PHAT), quality of life questionnaire (EQ-5D-5L) and the Lower Extremity Functional Scale (LEFS), and rated their satisfaction level on a scale from 0 to 100%. Local tenderness on the ischial tuberosity and maximum passive hip flexion were measured on both limbs. Maximum isokinetic knee flexor muscle strength was measured bilaterally using a dynamometer.

**Results:**

The median (range) PHAT, EQ-5D-5L and LEFS score were 78.8/100 (54.6–99.8), 0.94/1 (0.83–1) and 88.75/100 (61.25–100). The median satisfaction was 100% (90–100%). Only one patient felt discomfort when the ischial tuberosity was palpated. Neither maximum passive hip flexion nor maximum isokinetic flexor muscle strength differed between the operated and non-operated side (*P* > 0.58). Clinical scores did not correlate with the leg symmetry index of knee flexor muscle strength (Spearman’s rho < 0.448, *P* > 0.125). There were no tendon re-ruptures, or postoperative sciatic radiculopathy, at the time of follow-up.

**Conclusions:**

The modified extra-anatomical anchor placement resulted in good clinical and functional outcome of surgical repair of acute proximal hamstring rupture. Especially the absence of postoperative pain while sitting and the comparable muscle strength to the contralateral side is promising.

**Clinical trial registration:**

ClinicalTrials.gov Identifier: NCT04867746, registered.

## Introduction

Proximal hamstring rupture can affect trained athletes as well as the general population. For instance, the prevalence of proximal hamstring rupture in sports such as soccer, American football, baseball or track and field ranges from 5 to 40% and may depend on hours of sport exposure [1–5]. The most common injury mechanism is a simultaneous knee extension with hip flexion [6]. In adults, the conjoint tendon is most commonly involved in the rupture with or without a combination with the semimembranosus muscle [7]. There is a general consensus outlined in reviews that in case of complete rupture of all three tendons (i.e., semitendinosus, semimembranosus and biceps femoris muscles) or retraction (i.e., shortening) of at least two tendons by more than 2 cm, surgical treatment has a superior outcome than conservative treatment [6, 8–13] although there is no literature on the conservative management of older people with hamstring ruptures. Moreover, the current evidence suggests that the injured muscle should be reinserted on the original anatomical footprint in case of surgical refixation thereby closely restoring the natural anatomy [14–17]. The anatomical origin of the hamstring muscles on the ischial tuberosity is well known [18]. The semimembranosus muscle attachment is located more proximal and lateral than the attachment site of the conjoint tendon of the long head of the biceps femoris muscle and the semitendinosus muscle [13, 18, 19].

Some undesirable postoperative functional and clinical outcomes have been reported. Postoperative pain when seated has been frequently reported as the most common postoperative complaint and is seen in up to 61% of cases [19] with a mean pain score while seated of 64 (visual analogue scale, range 0–100) [20]. The ischial tuberosity—the anatomical origin of the hamstring muscles—is the point of maximal pressure and force transfer from the upper body on the seat surface while sitting [19, 21, 22] resulting, for instance, in irritation and/or pain in athletes after a first longer bike ride at the beginning of a cycling season [23]. Patients after anatomical proximal hamstring refixation onto the ischial tuberosity may experience pain due to the implant positioning, scar tissue or thickening of the tendon at the origin. Because people in developed countries spend more and more time sitting [24], addressing the complaint of pain while sitting in patients after proximal hamstring rupture is critical.

In addition, a decrease in maximum strength of the knee flexors has been described and accepted as an inevitable outcome [25]. This reported knee flexor strength deficit ranges from 12 to 15% [13, 26] to of up to 26% [16]. However, the existing literature on hamstring repair includes younger patients and/or athletes [13], and evidence for the outcome of hamstring repair in older patients who are not athletically ambitious are largely lacking. Because the capacity to produce force depends on the length of a muscle, proper muscle tensioning during surgical muscle repair is important. The force–length relationship of a muscle [27] implies that slight pre-tensioning of the muscle—achieved by minor stretching—can optimize its power output [27–30]. Hence, by pre-tensioning the injured muscle the surgeon can influence postoperative force capacity of the repaired muscle and hence its maximum muscle strength capacity as well as the muscle length at which this strength occurs.

Based on these considerations, we aimed to improve the current surgical technique by

- refixing the hamstring tendons proximal and lateral of their anatomical origin to pre-tensioning the muscle and hence.
- moving the insertion site of the tendons—especially of the conjoint tendon—away from the region loaded while sitting.

**Fig. 1:**
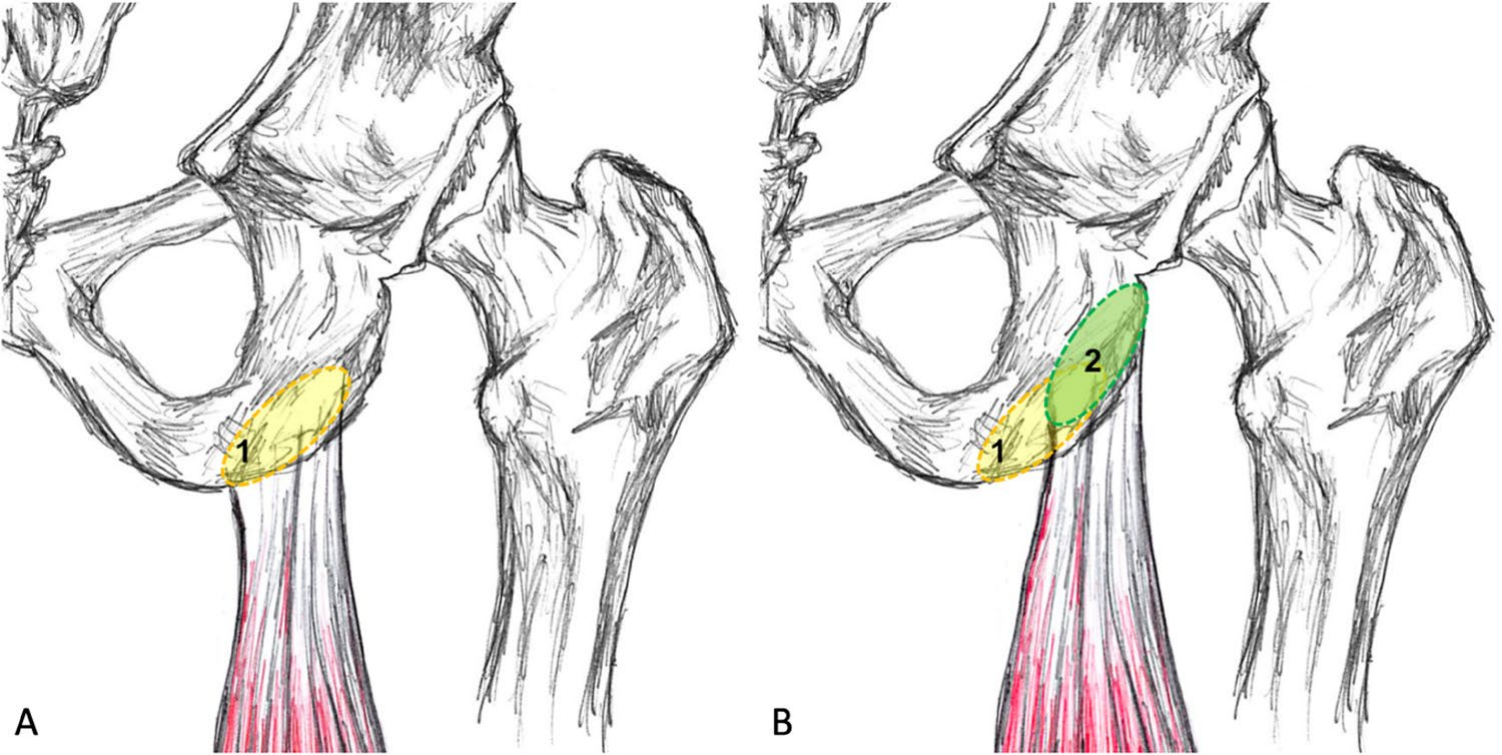
Illustration of the modified anchor placement. **A** anatomical attachment of proximal hamstring tendon on the ischial tuberosity of the right hip (1, yellow area); **B** modified, 10 to 15 mm (depending on the patients’ anatomy) more proximal and lateral attachment of both tendons (2, green area). Note that this anchor placement does not involve anchor placement on the caudal portion of ischial tuberosity

Combining these two modifications results in a modified extraanatomical insertion point on the supero-lateral side of the ischial tuberosity (Fig. 1). The objective of this study was to describe this modified surgical technique, to report clinical and functional outcome and patient satisfaction 1 to 6 years after proximal hamstring muscle repair with modified surgical anchor placement and to determine the association among these parameters in older patients with acute hamstring tendon rupture. We hypothesized that the modified anchor placement will result in good clinical outcome (low pain when seated, no palpation tenderness, no complications, high patient satisfaction), that the range of motion of the passive hip flexion, isokinetic muscle strength and thigh girth in the injured leg are comparable to those in the contralateral uninjured side and that clinical scores correlate with side-to-side differences in isokinetic muscle strength.

## Methods

Overall, 17 older patients were surgically treated within 4 weeks after acute proximal hamstring rupture between 2014 and 2019 at a single hospital by a single senior surgeon and all received the extraanatomical anchor positioning procedure. All patients were contacted retrospectively at the time of the study (November 2020 to June 2021) by the administrative personnel of our clinic and asked to participate in the follow-up examination. Inclusion criteria were age > 18 years and surgical repair with modified anchor placement surgically treated proximal hamstring ruptures between 2014 and 2019. Exclusion criteria were revision surgery within 6 months before testing on the ipsilateral knee and hip, injury and surgical procedures of the contralateral knee and hip within the last year, and inability to provide informed consent. The study was approved by the regional ethics committee. All participants signed informed consent prior to participation.

Of the 17 patients treated, 13 agreed to participate. One patient was not interested in the follow-up assessment because he was highly satisfied with the result of the operation. One patient only locomoted with an electric wheelchair, had just suffered a COVID-19 infection and was medically incapacitated to participate at the follow-up examination. One patient had severe acute rheumatic disease and one patient could not be reached via phone, email, or postal service. Two patients were only able to participate at specific dates because of personal and professional obligations and hence were allowed to participate before reaching the 24-month follow-up time (11 and 21 months after surgery, respectively).

### Surgical technique

An intubation or spinal anaesthesia was used, and a preoperative single shot of antibiotics with Cefazolin 2 g was intravenously administered according to the hospital standards. The patient was positioned prone with 60° hip flexion and simultaneous 60° knee flexion. Special care was taken to carefully support the patient’s body by padded surgical cushions to prevent a build-up of decubitus. A sterile gaze was precisely taped to fully cover the rima ani. A self-adhesive, sterile drape was then glued around the ipsilateral gluteus and the posterior part of the proximal thigh, to allow sufficient surgical exposure. The lower leg was fully covered with sterile stockinet and taped shut. The entire surgical aperture was then covered with an antimicrobial Ioban foil (3 M™ Ioban™ Steri-Drape™, 3 M™, MN, USA). The skin incision was performed strictly in the gluteal sulcus. Under a constant haemostasis with bipolar forceps, the incision was deepened to the deep fascia, which was displayed and horizontally incised. The caudal border of musculus gluteus maximus was identified and moved proximally. Medially and caudally from it, the hamstring fascia was vertically incised. The exposure was held in place with Langenbeck retractors and medially as well as laterally around ischial tuberosity placed Hohmann retractors. The sciatic nerve was not specifically searched for but great care was taken not to damage or compress it when placing the retractors.

**Fig. 2:**
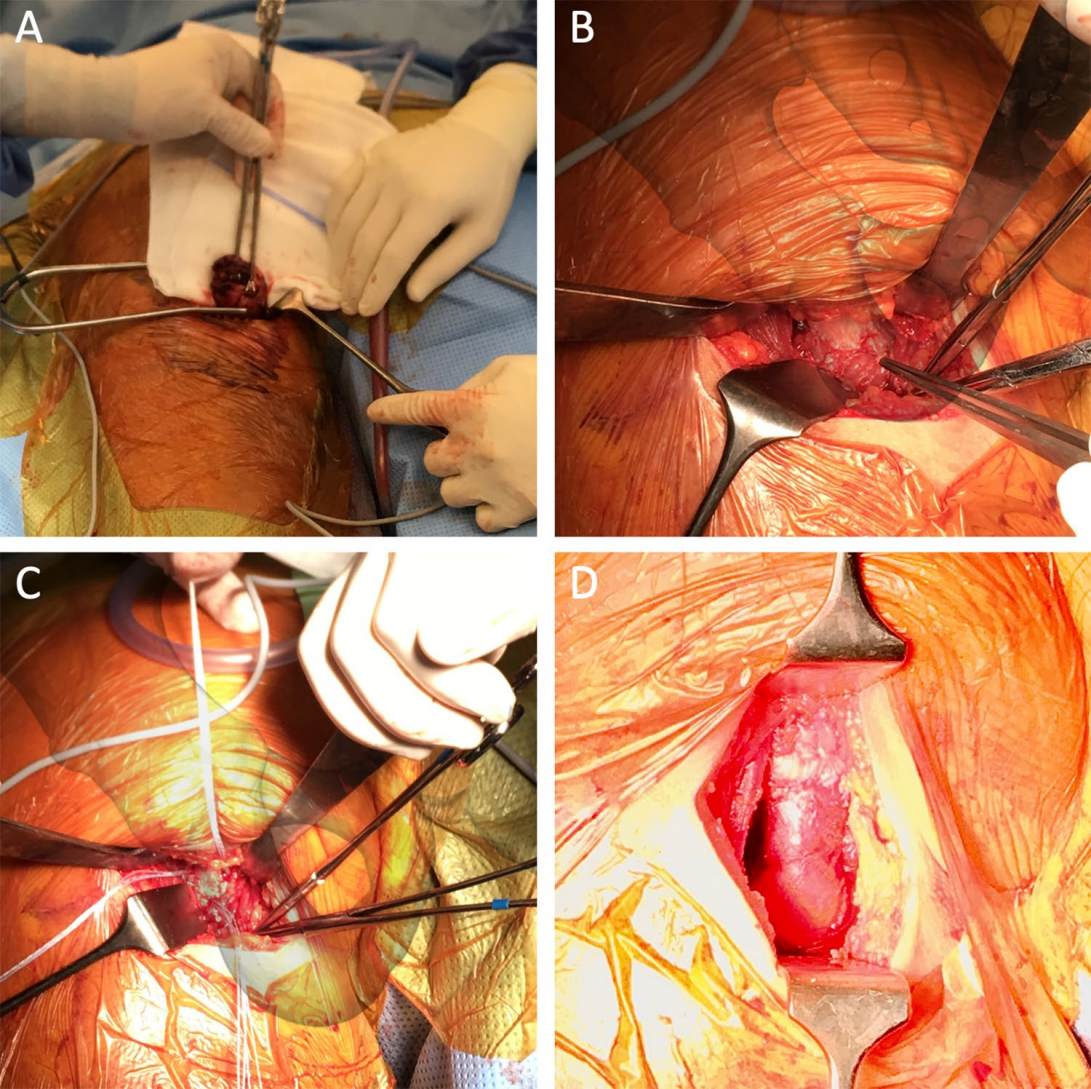
Intraoperative photographs. **A** torn proximal hamstring tendon is held with Kocher forceps; **B** debrided proximal and lateral part of ischial tuberosity just before anchor placement; **C** modified anchor placement with two anchors proximally and one distally; **D** completely refixed and adapted hamstring tendon. For illustrative purposes, we have added pelvic bone projections

The torn proximal hamstring tendons were identified, and a surgical debridement was performed. Subsequently, the hamstring origin on the ischial tuberosity was localized. We projected the modified reinsertion point 10 to 15 mm (depending on the patients’ anatomy) more lateral and proximal of the ischial tuberosity. This location was thoroughly debrided using a Luer forceps and three Arthrex BioComposite Corkscrews (FT 5.5 mm × 14.7 mm anchors, Arthrex, FA, USA) were introduced into the bone. Two of these were placed proximally and one distally. The non-resorbable FibreWire anchor-threads (FibreWire, Arthrex, FA, USA) were tightly sutured through the full thickness of the hamstring tendon using a Baseball-Stitch-Technique from proximal to distal. The armed tendon was then compressed using a Pulley-Method against the debrided bone and the anchors. After tying the surgical square knots, the remaining FibreWire was cut away (Fig. 2). Then, the wound was irrigated multiple times with Ringer-Lactate and the haemostasis was completed. The deep fascia of gluteus maximus musculature was sutured. The closure of the skin was subcutaneously performed with single sutures and the dermis itself with an intradermal resorbable suture and steri-strips. The wound was then covered with watertight plaster.

Postoperatively, the patients were allowed to partially bare weight (15 kg) and were constantly wearing a knee brace (Hinged Knee Brace, DonJoy, TX, USA) allowing knee extension up to 30° knee flexion angle for 8 weeks after the surgery. Hip flexion was not mechanically limited, but the patients were advised not to flex the hip more than 60° for 8 weeks after the surgery. First postoperative mobilisation took place under the control of a physiotherapist, and the patients were discharged when mobilisation and walking stairs with crutches was performed independently and safely. The supervised physiotherapy continued after hospital discharge. Initially, only procedures for muscle relaxation were allowed. After 4 weeks, isometric muscle strengthening and patellar mobilisation was introduced. Stretching of the hamstrings was prohibited for 4 months postoperatively. A daily use of thromboembolic prophylaxis with Deltaparin natrium (M.R. 4000–6000 D) (5000 UI s.c.) was prescribed for 8 weeks. Stitch removal was not necessary because of the use of a resorbable intradermal skin suture. Clinical follow-up was performed at 6 to 8 weeks postoperatively.

### Clinical and functional outcome assessment

At the single follow-up study visit, all patients completed the Perth Hamstring Assessment Tool (PHAT) [14, 31], a quality of life questionnaire (EQ-5D-5L) [32] and the Lower Extremity Functional Scale (LEFS) [33]. The PHAT assessment tool is a quick and reliable tool for measuring and comparing interindividual results in patients after a surgical treatment of a proximal hamstring rupture [31]. Additional to the over-all PHAT score, two of the PHAT-subscores (visual analogue scale (VAS): VAS when sitting, VAS at rest; 0–no pain; 100–maximum pain) were also included in further analyses. The EQ-5D-5L and LEFS scores are widely used cost-effective and simple methods for assessing the subjective perception, impairment and functionality of lower extremity in daily life after surgery [32, 33]. Additionally, patients were asked to rate their subjective satisfaction with the outcome of the surgery on a scale from 0 to 100% (0%–not satisfied at all; 100%–extremely satisfied).

A physical examination of both limbs of the patients was performed by a senior orthopaedic resident. Patients were asked to lie in prone position on a flat examination table. To assess local tenderness, the ischial tuberosity was palpated, and patients were asked whether they felt pain (yes/no) [34]. Patients were asked to turn to a supine position, and maximum passive hip flexion of both hip joints was examined [34–36]. Two measurements were made for each limb: maximum hip flexion with simultaneously flexed knee and maximum hip flexion with knee in full extension [9]. Hip flexion angles were measured with a goniometer centred on the previously palpated lateral tip of the greater trochanter.

Maximum isokinetic muscle strength of the knee flexors and extensors was measured for both limbs using an isokinetic dynamometer (Biodex Medical Systems 4 Pro, Mirion Technologies, GA, USA) by a trained movement scientist. The reliability, validity and accuracy of this system has been previously reported [37]. Patients were seated on the dynamometer, asked to freely test and familiarize with the knee movement permitted by the dynamometer. Individual maximal knee flexion and extension were set and registered on the dynamometer. Then, patients were asked to perform a series of five knee flexion and extension cycles at a velocity of 60°/s [37] using the previously determined maximum range of motion. After a break of 30 s, a second series of five cycles was executed. The maximum knee flexion torque of both series was used for each side, normalized to body mass (Nm/kg), and used for further analysis. Thigh girth was measured for both limbs 10 cm proximal of base of the patella using a tape measure.

### Statistical analysis

All study related data were collected and managed using REDCap electronic data capture tools hosted at our institution [38, 39]. All analyses were performed in SPSS Version 25 (IBM Corporation, Amonk, NY, USA). Significant differences in range of motion, muscle strength and thigh girth between the affected and the contralateral side were detected using Wilcoxon signed ranks tests. The limb symmetry index (LSI) was calculated as the maximum muscle flexion torque of the affected limb divided by the value for the unaffected limb multiplied by 100. An LSI of 90–110% can be considered as normal [40]. Spearman’s rank correlation coefficients were used to detect a potential correlation between the clinical scores and the LSI.

## Results

Of the 13 participants, 8 were females and 5 were males. The median age at follow-up was 64.2 years (range 52.1–80.4 years). The median time between trauma and surgery was 14 days. At follow-up, participants had a median body mass index of 28.5 kg/m^2^ (range 23.5–45 kg/m^2^). In seven patients the right side was injured, and in six patients the left side was injured. Twelve patients had a total (semitendinosus and biceps conjoint tendon and semimembranosus tendon) and one patient a partial (semitendinosus and biceps conjoint tendon) proximal hamstring rupture. The median time to follow-up assessment was 46.2 months (range 11.2–75.1 months) after surgery.

### PHAT, EQ-5D-5L, LEFS, subjective satisfaction and local tenderness

Median scores of clinical questionnaires (PHAT, EQ-5D-5L, LEFS) are shown in Table 1. All patients were very to extremely satisfied with the result of the surgery with a median subjective satisfaction rate of 100% (range 90–100%). Only one patient indicated local tenderness during palpation of the ischial tuberosity of the injured side (incidence of 7.7%). None of the patients experienced local tenderness on the contralateral side.

**Table 1:** Median (range) of the clinical scores and limb symmetry index (LSI) of the knee flexor strength, as well as Spearman’s correlation coefficients between clinical scores and LSI

Parameter	Median (range)	Correlation with LSI of flexor strength	
		Spearman’s rho	*P-*value
EQ-5D-5L	0.94 (0.83–1.00)	0.448	0.125
LEFS	88.8 (61.6–100.0)	0.192	0.529
*PHAT*			
Overall score	78.8 (54.6–99.8)	0.170	0.578
VAS when sitting	1.0 (0.0–8.1)	0.023	0.941
VAS at rest	0.0 (0.0–4.2)	0.075	0.808
LSI of flexor strength (%)	95.6 (71.7–135.6)		

*EQ-5D-5L* EuroQol-5 dimension—5-level questionnaire, *LEFS* lower extremity functional scale, *PHAT* Perth Hamstring Assessment tool with two sub-scores on pain on a visual analogue scale (VAS: 0–no pain; 10–maximum pain) when sitting and at rest, *LSI* limb symmetry index

### Range of motion

**Fig. 3:**
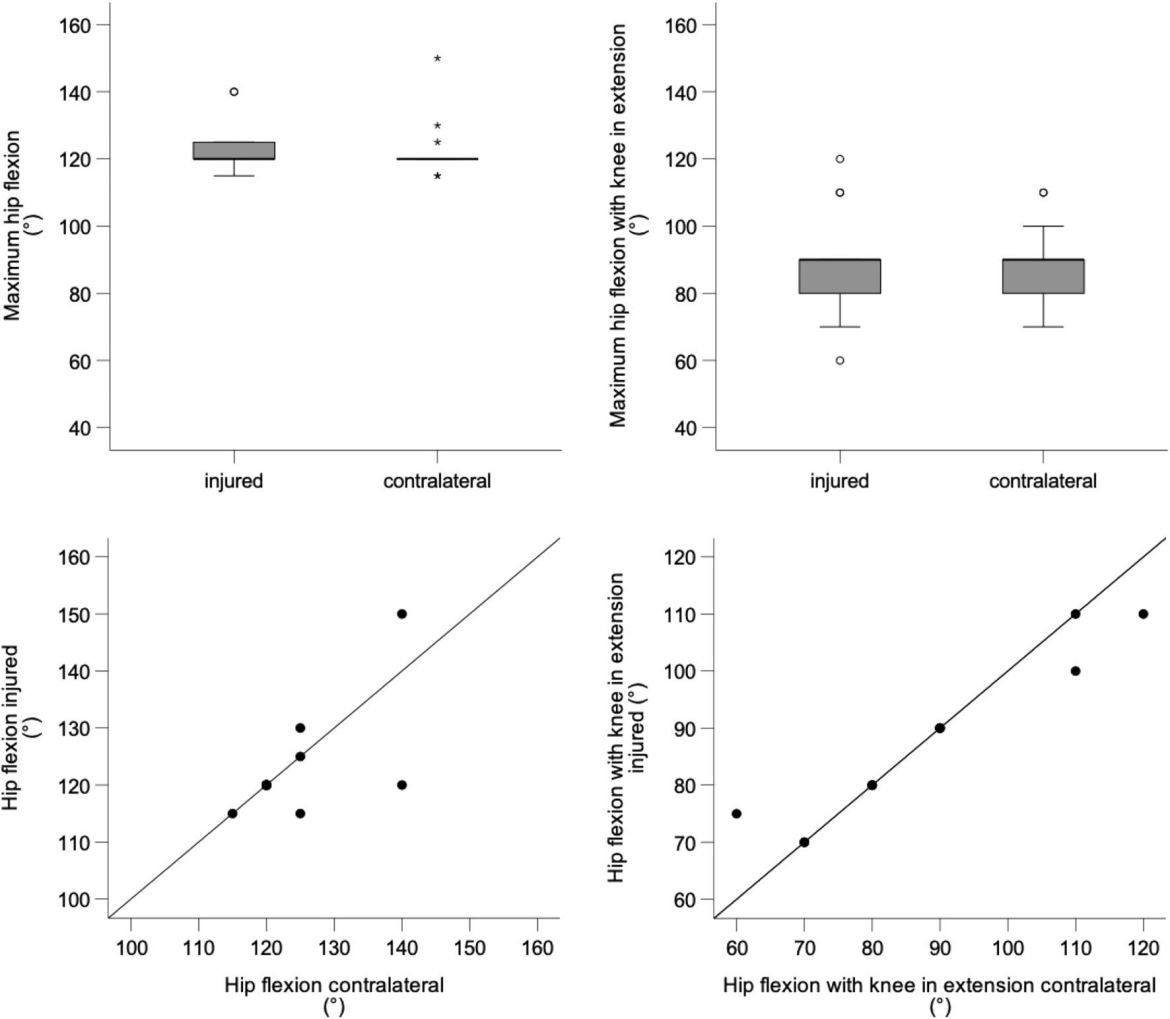
Box plots showing the median and interquartile range of the maximum passive hip flexion with the knee in flexion (top left) and maximum passive hip flexion with the knee in extension (top right) of the injured and contralateral leg; scatter plots showing the relationship between maximum hip flexion of the injured and contralateral leg with knee in flexion (bottom left) and knee in extension (bottom right)

Patients had a median maximum passive hip flexion with the knee in flexion of 120° (range 115–140°) on the operated side and 120° (range 115–150°) on the contralateral side (*P* = 0.581; Fig. 3). Patients had a median maximum passive hip flexion with the knee in extension of 90° (range 60–120°) on the operated side and 90° (range 70–110°) on the contralateral side (*P* = 1.000). Patients with greater values on the operated side had greater values on the contralateral side (Fig. 3).

### Isokinetic muscle strength

**Fig. 4:**
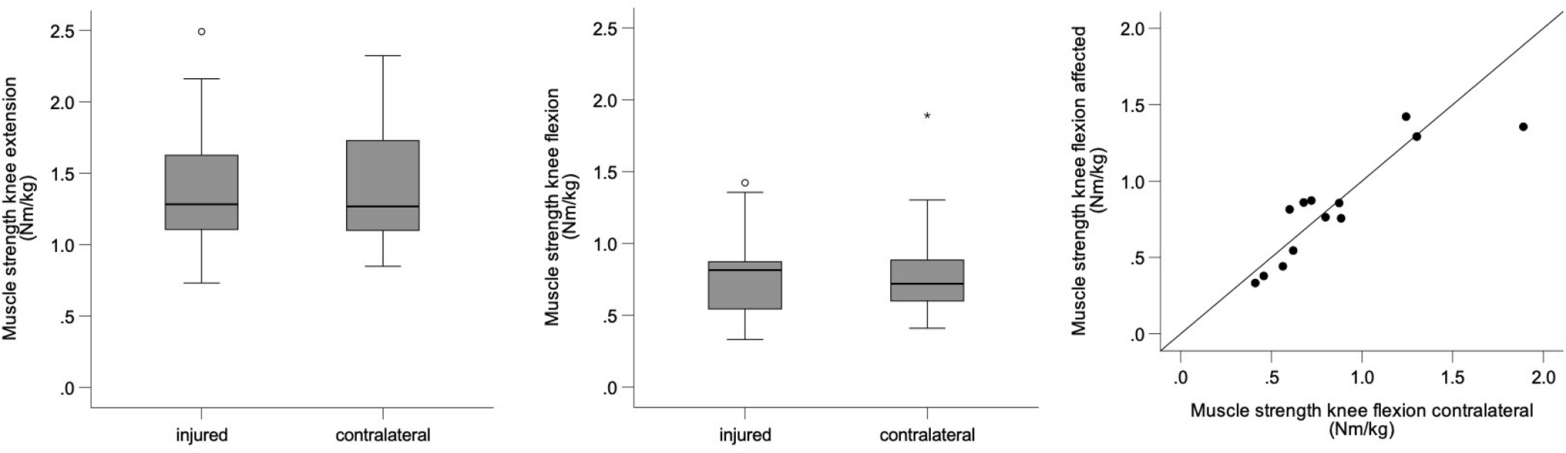
Box plots with median and interquartile range of the isokinetic knee extension torques of the injured and contralateral leg (left) and isokinetic knee flexion torques of the injured and contralateral leg (middle) and scatter plot showing the relationship between knee flexion muscle strength of the injured and contralateral leg (right)

**Fig. 5:**
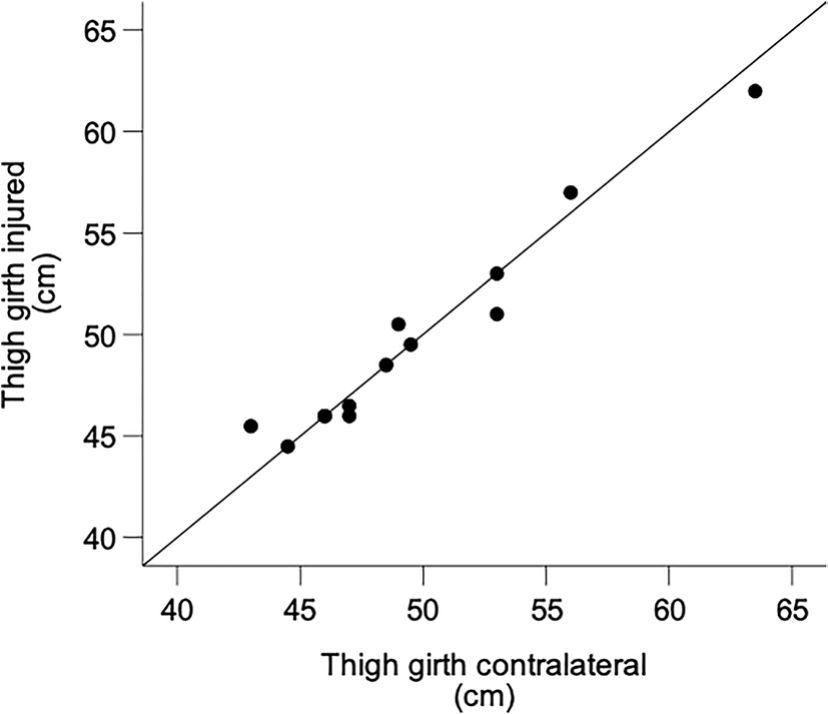
Scatter plot showing the relationship between thigh girth of the injured and contralateral leg

Patients had a median maximum flexor muscle strength of 0.82 Nm/kg (range 0.33–1.42 Nm/kg) on the operated side and 0.72 Nm/kg (range 0.41–1.89 Nm/kg) on the contralateral side (*P* = 0.807; Fig. 3). The median LSI of maximum flexor muscle strength was 95.6% (range 71.7–135.6%) corresponding to a median muscle strength deficit of 3.5% in the operated side. Thigh girth did not differ between the operated and the contralateral leg (operated: median 48.5 cm, range 43–63.5 cm; contralateral: median 48.5 cm, range 44.5–62.0 cm; *P* = 1.000). Patients with greater muscle strength and thigh girth on the operated side had greater values on the contralateral side (Figs. 4 and 5).

### Association between clinical scores and LSI

We found no significant correlations between any of the clinical scores (PHAT, EQ-5D, or LEFS) and the LSI in flexor muscle strength (Spearman’s rho < 0.448, *P* > 0.125; Table 1).

### Postoperative complications

One patient had a superficial wound infection, which was successfully surgically treated and healed without any further problems. One patient had local skin-dehiscence, which was excised and newly sutured. There were no tendon re-ruptures and no postoperative sciatic radiculopathy at the time of follow-up.

## Discussion

The objective of this study was to describe a modified surgical technique, to report clinical and functional outcome and patient satisfaction around 2 to 5 years after acute proximal hamstring muscle repair with modified surgical anchor placement, and to determine the association among these parameters in older patients. Our results confirmed our hypothesis that isokinetic muscle strength and thigh girth in the injured leg are comparable to those in the contralateral uninjured side. Contrary to our secondary hypothesis, clinical scores did not correlate with side-to-side differences in isokinetic muscle strength assessed as LSI of maximum knee flexion torque. Moreover, patient satisfaction was very high, and we observed few complications. These results suggest that the modified surgical anchor placement is a promising technique with good to excellent clinical and functional outcome.

The subjective satisfaction rate in our patients was extremely high and comparable to non-modified hamstring refixation (98.8 vs. 97% [19]). Similarly, the PHAT score was comparable to what was reported for patients after other surgical refixation methods (78.8 vs. 74.1 [41], 79.8 [42] and 79.9 [43]). The questions with the lowest points in the PHAT were number of minutes without discomfort while driving a car, number of minutes without discomfort while running and description of the current level of activity. The patients in our study were older patients with an age range of 52–80 years. One older female patient did not own or drive a car so she marked the answer “zero minutes without discomfort while driving a car”. Similarly, some patients did not participate in any sports and also did not run, and hence marked “zero minutes without discomfort while running” and that they were not able to play sports. These results suggest that this questionnaire may not be well suited for a generally less active population.

The importance of reducing the high incidence of feeling pain while sitting [19, 44] will become even more critical because the Western population spends increasing times sitting [24]. Moreover, chronic pain is the second most frequent reason for disability to work [45], and return-to-work programs are challenging [46, 47]. Most patients with proximal hamstring rupture are athletes and/or middle-aged persons [25] with a reported age around 42 to 47 years [19, 20, 44]. Hence, any surgical treatment should be designed to ensure about 20 more years of productive occupational work, which will usually be mostly in seated postures. In our study, only one of thirteen patients reported pain while sitting and local tenderness of ischial tuberosity corresponding to an incidence of 7.7%, which is markedly lower than the incidence of up to 61% incidence [25] reported in the literature. The treatment of proximal hamstring rupture has changed and progressed rapidly over the past few years. A systematic review reported superior outcome of surgical versus conservative therapy although evidence for conservative therapy is low and no study provided a direct comparison [13]. Moreover, consensus has been established that the likelihood of good postoperative result will increase with reducing the time from injury to the operation [26, 48]. These results emphasize the potential of further developing surgical techniques to achieve better clinical outcome as observed in our study.

We observed no measurable difference in maximum passive hip flexion between the operated and the contralateral hip, both with the knee in flexion as well as with the knee in extension. We also did not observe a difference in postoperative isokinetic maximum muscle strength between the operated and contralateral hamstring muscles. We noted two outliers in our measurements. One patient had an LSI of 135.6% corresponding to a 35.6% greater maximum flexor muscle strength on the operated side than on the contralateral side. Interestingly, this patient did not participate in any prescribed physiotherapy sessions but is a physiotherapist by training. We speculate that based on her background she was well aware of the importance of strength exercises and may have completed more rigorous training for a longer period than other patients. However, because we did not record or monitor completed physiotherapy sessions in this retrospective cross-sectional study, this remains speculation. Nonetheless, these results clearly show that hamstring muscle strength after this modified surgery may not only be comparable to but may far exceed the hamstring muscle strength of the contralateral side.

The other outlier had an LSI of 71.7%, corresponding to a 28.3% lower maximum flexor muscle strength on the operated side than on the contralateral side. This result was particularly surprising because this patient was still a semi-professional track and field athlete at the time of the follow-up visit. This patient had the highest maximum flexor muscle strength with noticeably weaker hamstring muscles on the operated compared to the contralateral side. This finding was surprising also to the patient because he had not experienced any functional limitations, had fully returned to sport, and was extremely satisfied with the result of the surgery.

In contrast to our study, reduced hamstring muscle strength after hamstring repair is still a common outcome. In some studies (all including younger cohorts than ours), patients were asked to subjectively estimate their hamstring muscle strength after a proximal hamstring refixation. The results showed a subjectively estimated residual strength of more or equal to 75% [19, 44], which must be considered unsatisfactory. In other words, the patients may subjectively estimate a muscle strength deficit of up to 25%, even though the previously reported objectively measured strength deficit may be only around 15% at 12 months after surgery [13, 26, 49]. This discrepancy emphasizes the need for an objective, quantitative, and, most importantly, comparable assessment of postoperative hamstring muscle strength between studies, as recommended by Reza et al. [50] and Fouasson-Chailloux et al. [49]. The overall deficit of peak flexor strength in the affected leg achieved in our patients (3.5%) is well below the 15% reported in the review by Fouasson-Chailloux et al. [49].

The very positive outcome of this modified surgical technique is likely linked to the modified positioning of the anchors. Overall, these promising results show that the presented technique is a feasible option for surgical treatment of acute proximal hamstring ruptures in older patients. These results need to be confirmed in clinical trials involving more patients.

### Strength and limitations

In this study, we not only reported on patient reported outcome in patients after hamstring repair but also included objective quantitative functional parameters. Combining subjective satisfaction results and clinical scores with muscle strength measurements provided important insight into the outcome of a modified surgical technique. Because hamstring rupture is not a very common injury, only 17 patients were treated between 2014 and 2019 of which 13 volunteered to participate in this retrospective case series. Our patients were older than the populations in previous studies, were not athletically ambitious, and had a large variation in body mass index. Hence, the results of our study may not be readily transferrable to younger populations or athletes. As all patients at our clinic were treated with the modified procedure, we were not able to include a control group and hence we compared our results with those reported in the literature. The fact that all patients were treated by the same surgeon can be seen as strength and limitation as the treatment in this study was consistent but the influence of surgeon remains unknown. Outcome parameters were not assessed preoperatively because of the acute nature of the injuries. Nonetheless, the data presented here are promising and provide initial evidence that the described modified surgical technique produces good clinical and functional outcome in older patients.

## Conclusion

After surgical repair of acute hamstring rupture using an off-anatomical insertion cite, older patients were very satisfied and had good clinical and functional outcome. Compared to the contralateral side, patients had comparable passive range of motion, muscle strength and thigh girth on their operated side. These results suggest that the modified surgical technique is feasible and results in excellent outcome that may be similar or even superior to those of established surgical techniques in older patients after acute hamstring rupture.

## Data Availability

The data that support the findings of this study are available from the corresponding author (KS)], upon reasonable request.
